# Global trends in research of high-throughput sequencing technology associated with chronic wounds from 2002 to 2022: A bibliometric and visualized study

**DOI:** 10.3389/fsurg.2023.1089203

**Published:** 2023-02-22

**Authors:** Hao Meng, Yu Peng, Pinxue Li, Jianlong Su, Yufeng Jiang, Xiaobing Fu

**Affiliations:** ^1^ Research Center for Wound Repair and Tissue Regeneration, Medical Innovation Research Department, The PLA General Hospital, Beijing, China; ^2^ School of Medicine, Nankai University, Tianjin, China

**Keywords:** chronic wound, high-throughput sequencing, bibliometric analysis, visualized study, microbial ecology, wound healing

## Abstract

**Background:**

Chronic wounds are a complex medical problem. With the difficulty of skin healing, the microbial ecology of chronic wounds is an essential factor affecting wound healing. High-throughput sequencing (HTS) technology is a vital method to reveal the microbiome diversity and population structure of chronic wounds.

**Objective:**

The aim of this paper was to delineate the scientific output characteristics, research trends, hotspots and frontiers of HTS technologies related to chronic wounds globally over the past 20 years.

**Methods:**

We searched the Web of Science Core Collection (WoSCC) database for articles published between 2002 and 2022 and their full record information. The Bibliometrix software package was used to analyze bibliometric indicators and VOSviewer visualization analysis results.

**Results:**

Ultimately, a total of 449 original articles were reviewed, and the results showed that the number of annual publications (Nps) about HTS associated with chronic wounds has steadily increased over the last 20 years. The United States and China produce the most articles and have the highest H-index, while the United States and England have the largest number of citations (Nc) in this field. The University of California, Wound Repair and Regeneration and National Institutes of Health Nih United States were the most published institutions, journals and fund resources, respectively. The global research could be divided into 3 clusters as follows: microbial infection of chronic wounds, the healing process of wounds and microscopic processes, skin repair mechanism stimulated by antimicrobial peptides and oxidative stress. In recent years, “wound healing”, “infections”, “expression”, “inflammation”, “chronic wounds”, “identification” and “bacteria” “angiogenesis”, “biofilms” and “diabetes” were the most frequently used keywords. In addition, research on “prevalence”, “gene expression”, “inflammation” and “infection” has recently become a hotspot.

**Conclusions:**

This paper compares the research hotspots and directions in this field globally from the perspectives of countries, institutions and authors, analyzes the trend of international cooperation, and reveals the future development direction of the field and research hotspots of great scientific research value. Through this paper, we can further explore the value of HTS technology in chronic wounds to better solve the problem of chronic wounds.

## 1. Introduction

Chronic wounds on the body surface refer to wounds that have not healed and have no tendency to heal after three months of treatment (1, 2). The International Association for Wound Healing defines chronic wounds as those that cannot achieve anatomical and functional integrity through a normal, orderly and timely repair process. Chronic wounds will seriously affect the quality of life of patients and increase the high medical costs due to the need for repeated treatment (3, 4). The repair of chronic refractory wounds is an ancient and complex medical problem. With the improvement of global medical level, technology and cognition in recent decades, many achievements have been made in the repair of chronic wounds, and key points and potential methods for treating chronic wounds in the future have also been found (5, 6).

A complex wound microbial environment is an important reason for chronic wound persistence (7, 8). Infection is also an important pathogenic factor for sepsis and multiple organ failure in patients with chronic wounds and a major threat to the life of patients with chronic wounds (9–12). It is currently believed that the bacterial-host interaction may also lead to wound healing damage (8, 13). Clarifying the microbial environment of the wound is conducive to wound treatment (14). Although the traditional culture method is mature and reliable, only approximately 1% of the bacteria in the biological world can be cultured, which cannot fully reflect the real situation of the wound microenvironment (15). In recent years, high-throughput sequencing (HTS) technology has developed rapidly and become a new hotspot in wound microbial detection (16–18). HTS technology can analyze the genome of all microorganisms in the sample and can find many bacteria that cannot be cultured and are not affected by amplification preference (19). The identification results can be accurate to species, even strain level, effectively making up for the shortcomings of the culture method (20). It is of great significance to study the application status, research hotspots and future prospects of HTS technology in the field of chronic wounds.

## 2. Materials and methods

### 2.1. Data source and search strategy

**Figure 1:**
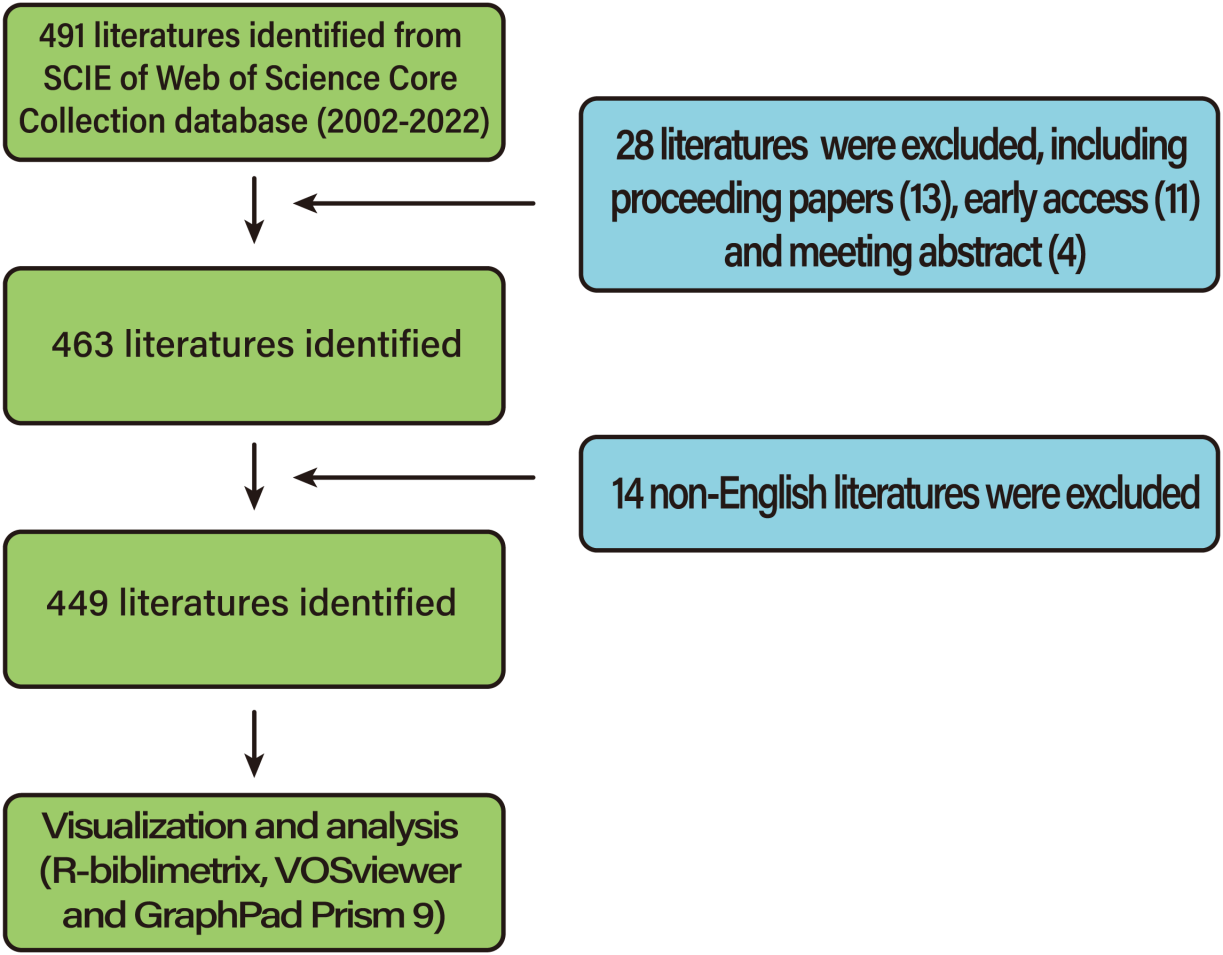
Flowchart of the screening process.

The database we selected is the Web of Science Core Collection. The data were downloaded from the Web of Science Core Collection on the same day as October 5, 2022. The search formula was TS = ((chronic wounds) OR (diabetes wounds) OR (chronic diabetic foot)) AND TS = ((16S rRNA) OR (16S rDNA) OR (metagenome) OR (metagenomics) OR (high-throughput sequencing) OR (sequencing)), and the search time range was from October 4, 2002 to October 4, 2022. A total of 491 articles were retrieved, excluding proceeding papers (13), early access (11) and meeting abstracts (4). In addition, 14 non-English articles were excluded, so ultimately, 449 articles were included in our study (Figure 1).

### 2.2. Data collection and cleaning

A total of 449 studies were exported in the form of all records and references, saved as the format of BibTex and Table delimited file. Coauthors (PY and MH) independently browsed and extracted all the data from these articles, manually deleted duplicate and wrong data, corrected spelling errors, and resolved all the divergences by discussing with experts to reach a final consensus. Finally, the corrected data were imported into the bibliometric package and VOSviewer for bibliometric analysis.

### 2.3. Bibliometric analysis

The techniques of bibliometrics analysis are presented in two categories: performance analysis and science mapping. In essence, performance analysis accounts for the contributions of the components under study, while scientific mapping focuses on the relationships between the components under study. Bibliometric performance analysis indicators usually include the number of publications (Np) and the number of citations without self-citation (Nc) (21, 22). In general, Np is used to measure the scientific research productivity of a country, institution or author, and Nc is used to measure the scientific research influence of a country, institution or author. Np and Nc are two main perspectives for evaluating the research level. The H-index is an evaluation index that links and balances Np and Nc (23, 24). It is increasingly used to evaluate researchers' academic contributions and predict future scientific achievements (25). H-index means that if a researcher has published H papers and each paper has been cited at least H times, then the researcher will have an H-index (26). In addition, the impact of a journal can be evaluated by the impact factor (IF) in the latest edition of the Journal Citation Report (JCR), which is the average number of citations of articles published by the journal in a year and can reflect the academic quality and impact of the journal (27).

### 2.4. Visualized analysis

For the visual analysis of the analyzed data, we used the Bibliometric package based on R language and the VOS viewer based on Java for analysis (11). The types of bibliometric functions include the main processes of scientific knowledge graph rendering, such as data import, format transformation, data cleaning and sorting, descriptive statistics, co-occurrence matrix establishment, data standardization, and graph rendering (22). The VOS viewer tool is useful at generating any type of text map. It can perform cooperative network analysis, co-occurrence analysis, citation analysis, document coupling analysis, and co-citation analysis (22).

## 3. Results

### 3.1. Trend of publication outputs

**Figure 2:**
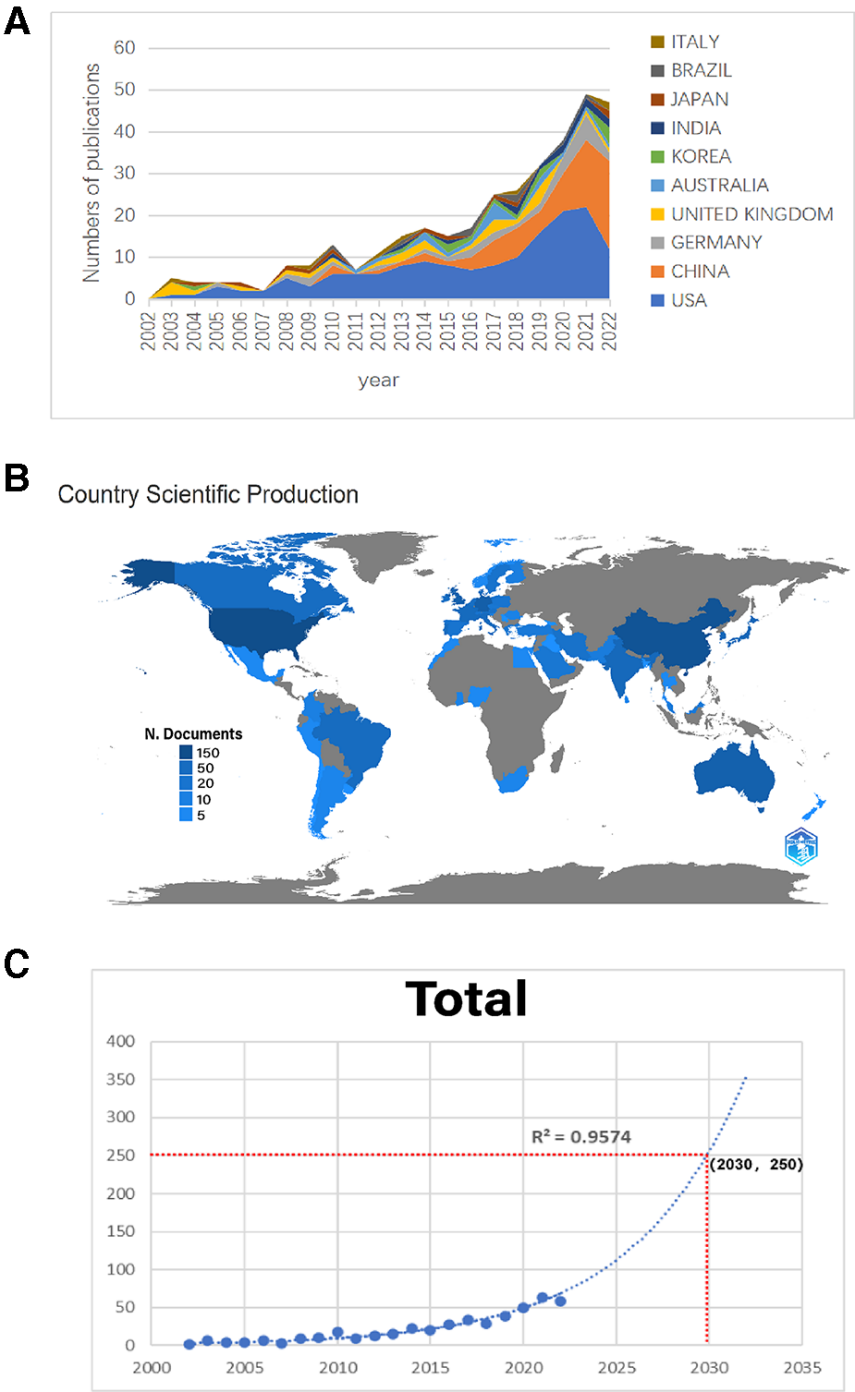
(**A**) The number of publications by year over the past 20 years. (**B**) Distribution of scientific production numbers between countries in the world map. (**C**) Curve fitting of the total annual growth trend of publications (*R*^2^ = 0.9574).

According to the retrieval criteria, a total of 491 articles were retrieved, except for proceedings papers (13 articles), early access (11 articles) and meeting abstracts (4 articles). In addition, 14 non-English articles were excluded, and a total of 449 articles were finally included in our study. As shown in Table 1, these 449 articles come from 292 sources, including journals and books. The annual growth rate was 22.51 percent. On average, each article was cited 36.64 times. Eleven authors participated in the single-author document, while 2,585 authors participated in the multiauthor document. There were 6.6 coauthors in each paper, and 20.49% were international coauthors. From 2002 to 2022, the number of global studies showed a steady growth trend year by year. In 2002, there was only one, while in 2022, there were 58. The majority of studies were published in 2021 (63,14.03%). The polynomial fitting curve of the annual trend of publication volume is given. The annual Np was significantly correlated with the year of publication, and the correlation coefficient R2 reached 0.9574. The number of published papers is expected to reach 250 by 2030 (Figure 2C). Taken together, these results indicate that HTS technology related to chronic wounds has attracted increasing attention from researchers and has reached a stage of rapid development.

**Table 1:** Descriptive data of retrieved information about HTS associated with chronic wound.

Description	Results
Timespan	2002:2022
Sources (Journals, Books, etc)	292
Documents	449
Annual Growth Rate %	22.51
Document Average Age	5.21
Average citations per doc	36.64
References	22,141
**DOCUMENT CONTENTS**	
Keywords Plus (ID)	1,833
Author's Keywords (DE)	1,352
**AUTHORS**	
Authors	2,585
Authors of single-authored docs	11
**AUTHORS COLLABORATION**	
Single-authored docs	12
Co-Authors per Doc	6.6
International co-authorships %	20.49
**DOCUMENT TYPES**	
article	359
article; early access	11
article; proceedings paper	8
review	71

### 3.2. Distribution of countries/regions and institutions

**Figure 3:**
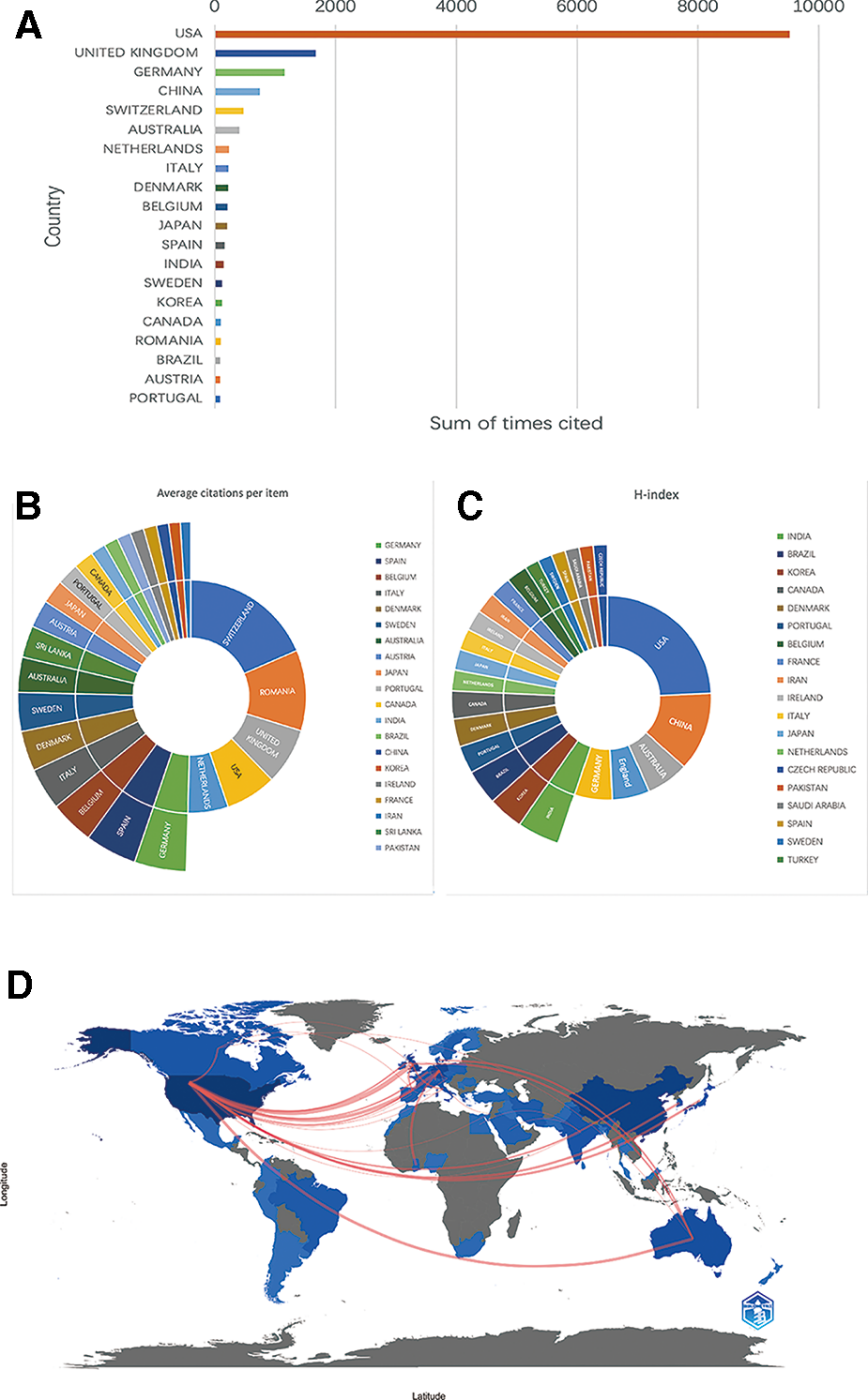
(**A**) The top 20 countries/regions of total citations. (**B**) The top 20 countries/regions of the average citations per paper. (**C**) The top 20 countries/regions of the H-index. (**D**) The geographical network map of cooperative relations between countries.

We rank the 10 countries/regions with high output for all authors according to their Np values (Figure 2A). The United States (Np = 156, 34.74%) and China (Np = 74, 16.48%) contributed 51.22% of the papers and were the main drivers of research in this field, followed by Germany (Np = 27, 6.01%), the United Kingdom (Np = 24, 5.35%) and Australia (Np = 15, 3.34%). The distribution of scientific production numbers between countries in the world map is shown in Figure 2B. The United States papers were cited 9,520 times, accounting for 58.39% of the total, followed by the UK (Nc = 1,672) and Germany (Nc = 1,152) (Figure 3A). For each type of citation, publications from Switzerland had the highest average number of citations (Ac), which was 157 times. Romania ranked second in average citations (Ac = 95), ahead of the United Kingdom (Ac = 66.88), the United States (Ac = 60.64) and the Netherlands (Ac = 61.76) (Figure 3B). In addition, the United States had the highest H-index (H-index = 33), followed by China (H-index = 17), Australia (H-index = 9), Germany (H-index = 8) and the UK (H-index = 8) (Figure 3C). Regarding publication ranking, the top 5 contributive institutions were listed here. The first was Michigan University (28 publications), followed by Stanford University (26 publications) and Miami University (26 publications). Kunming Med University and The Ohio State University were tied for third (20 publications). As seen from Figure 3D, the cooperative network map mainly existed in North America, Western Europe and East Asia.

### 3.3. Analysis of global leading journals, authors and funding sources

For the analysis of global leading journals, the results are shown in Table 2. *Wound Repair and Regeneration* (Np = 18, IF: 3.401) published the most papers on HTS technology for chronic wounds, followed by *Plos One* (Np = 12, IF: 3.752). The *International Journal of Wounds* (Np = 7, IF: 3.099) and *Frontiers in Cell and Infection Microbiology* (Np = 7, IF: 6.073) tied for third place. In terms of Nc, *Wound Repair and Regeneration* were the highest (Nc = 2,567), followed by *BMC microbiology* (Nc = 729), *Plos One* (Nc = 334), *Clinical Microbiology and Infection* (Nc = 240) and *International Journal of Wound* (Nc = 223). The H-index of *Wound Repair and Regeneration* ranked first (H-index = 18), followed by *Plos One* (H-index = 12). The H-indexes of *BMC Microbiology,* I*nternational Journal of Wound* and *Compendial Wound Clinical Research and Practice* were all 5, tied for third place.

**Table 2:** The top 10 most productive journals about HTS associated with chronic wound.

RANK	Journal	Np	Nc	H-index	IF (2022)
1	Wound Repair and Regeneration	18	2,567	12	3.401
2	Plos One	12	334	8	3.752
3	International Wound Journal	7	223	5	3.099
4	Frontiers in Celluar and Infection Microbiology	7	71	4	6.073
5	JCI Insight	6	103	4	9.484
6	BMC Microbiology	5	729	5	4.465
7	Wounds	5	60	5	1.441
8	Clinical Microbiology and Infection	4	240	4	13.31
9	Advance in Wound Care	4	106	4	4.947
10	Annals of Plastic Surgery	3	37	3	1.763

Referring to the analysis of global leading authors, the top 10 productive authors are listed in Table 3. They contributed 63 publications, accounting for 14.03% of the total number of papers. Both Li X and Wang Y contributed 9 productions, ranking first, followed by Malone M (Np = 7). Among the top 10 productive authors, four were Chinese, three were Australians, two were Americans and one was from Denmark. Li X (H-index = 7) had the highest H-index, and Grice Ea (H-index = 662) had the highest total citation frequencies, followed by Wolcott Rd (H-index = 628).

**Table 3:** The top 10 authors with the most publications about HTS associated with chronic wound.

RANK	Author	Country	Np	Nc	H-index
1	LI X	China	9	204	7
2	GRICE EA	United States	6	662	6
3	MALONE M	Australia	7	135	5
4	TANG J	China	6	110	5
5	WANG Y	China	9	101	5
6	WOLCOTT RD	United States	5	628	5
7	YANG M	Australia	6	99	5
8	YANG X	China	6	86	5
9	BJARNSHOLT T	Denmark	5	185	4
10	HU H	Australia	4	146	4

About analysis of global leading founding resources: National Institutes of Health (Np = 92) ranked in No.1, followed by United States Department of Health Human Services (Np = 92), National Natural Science Foundation of China (Np = 48), European Commission (Np = 20) and NIH National Institute of General Medical Sciences (Np = 18).

### 3.4. Bibliographic coupling analysis of country, journal and institution

**Figure 4:**
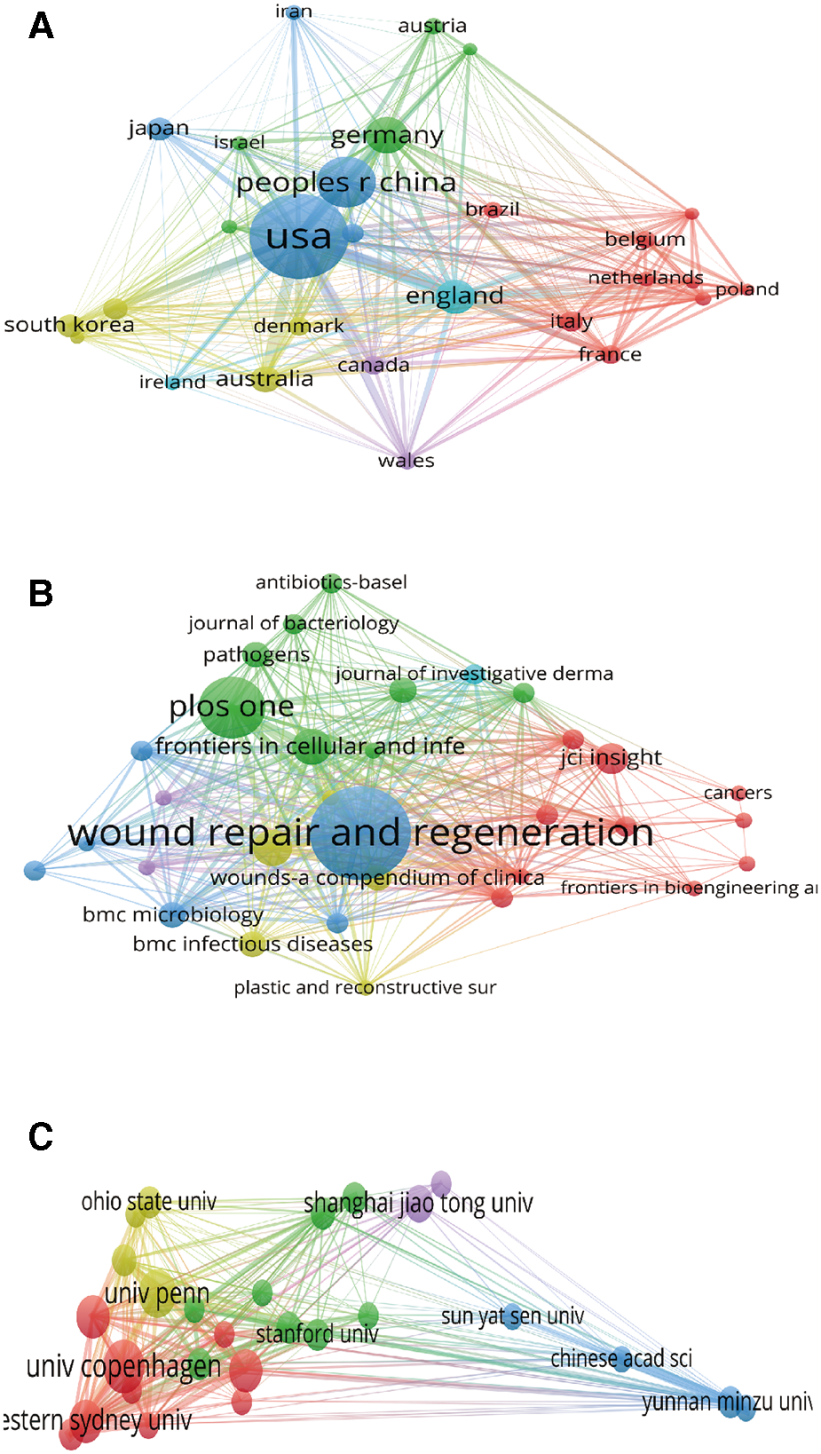
Mapping of bibliographic coupling analysis of HTS technology associated with chronic wounds. (**A**) Mapping of the 28 identified countries. (**B**) Mapping of the 32 identified journals. (**C**) Mapping of the 27 identified institutions.

Bibliographic coupling analysis is used to measure the relevance of institutions, journals and countries by analyzing the number of references cited to the same reference between them (28). Countries (defined as the minimum number of documents is 5) bibliographic coupling analysis in VOS viewer is presented in Figure 4A. A total of 28 countries meet the thresholds, among which the 5 with the strongest link strength were “United States (total link strength = 12,305)”, “England (total link strength = 5,493)”, “Germany (total link strength = 3,591)”, “Australia (total link strength = 3,154)”, and “France (total link strength = 2,548)”.

Journals (defined as the minimum number of documents is 3) bibliographic coupling analysis in VOS viewer is presented in Figure 4B. A total of 32 journals meet the thresholds, among which the 5 journals with the strongest link strength were “Wound Repair and Regeneration (total link strength = 914)”, “Frontiers in Cellular and Infection Microbiology (total link strength = 623)”, “International Wound Journal (total link strength = 588)”, “Plos One (total link strength = 551)”, and “BMC Microbiology (total link strength = 348)”.

A total of 27 institutions (defined as the number of published articles at least 5) were analyzed, among which the 5 with the strongest link strength were “Univ Penn (total link strength = 863)”, “Western Sydney Univ (total link strength = 831)”, “Ingham Inst Appl Med Res (total link strength = 716)”, “Yunnan Minzu Univ (total link strength = 706)”, and “Univ Copenhagen (total link strength = 641)” (Figure 4C).

### 3.5. Co-citation analysis of cited journals, authors and references

Co-citation analysis is a scientific mapping technique that assumes that publications that are frequently cited together are similar in topic. This analysis can be used to reveal the knowledge structure of a research area, such as its underlying topics. In a co-citation network, two publications are linked when they appear in another publication's reference list at the same time.

**Figure 5:**
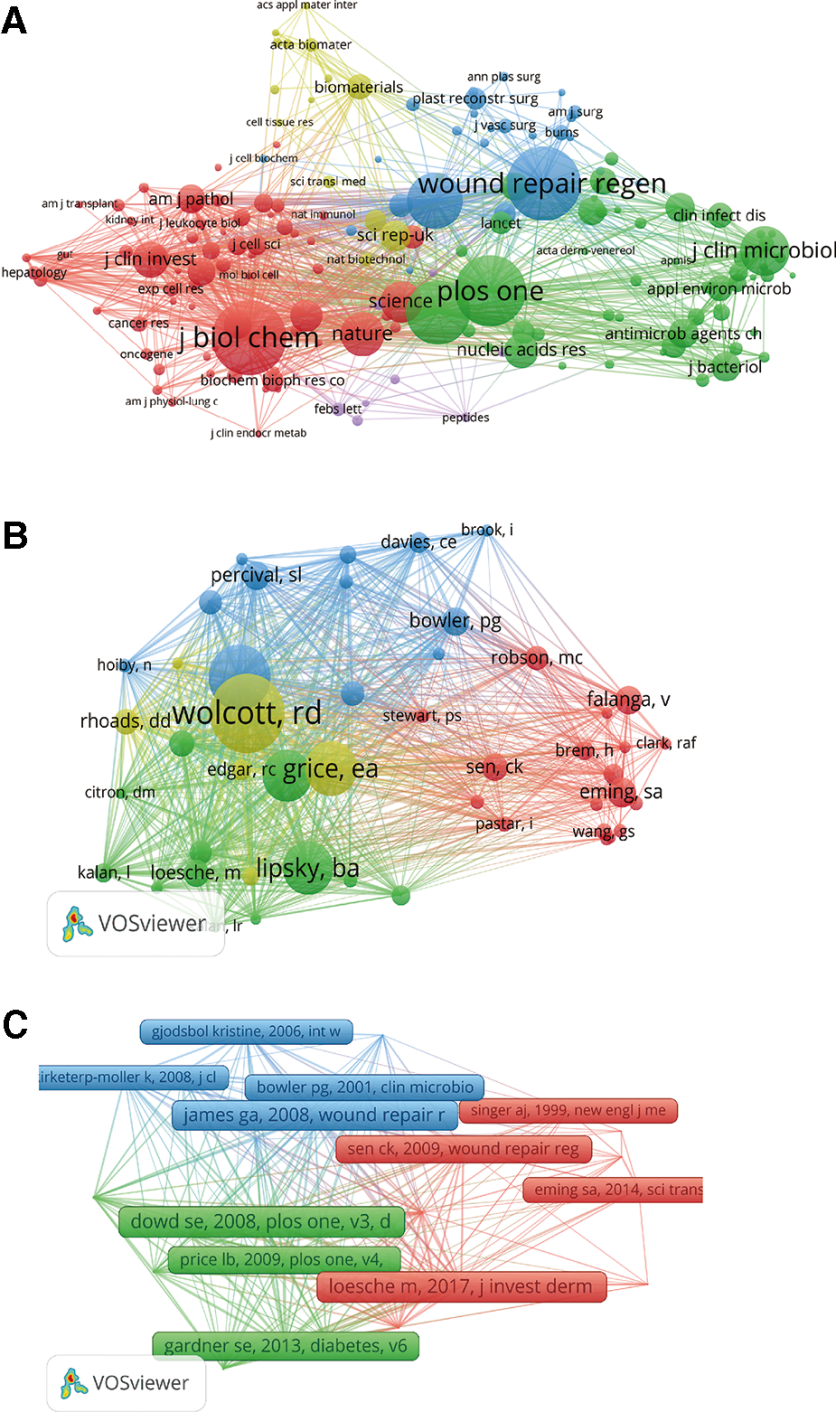
Mapping of co-citation analysis of HTS technology associated with chronic wounds. (**A**) Mapping of the 172 identified journals. (**B**) Mapping of the 48 identified authors. (**C**) Mapping of the 25 identified references.

When performing the co-citation analysis of cited journals on VOS viewer, we screened out the 30 references and journals cited above, and 172 of them were selected out of 4,453 journals. Sorted according to total link strength, the top five were *J Biol Chem* (total link strength = 31,615), *Plos One* (total link strength = 23,399), *P Natl Acad Sci United States* (total link strength = 22,616), *Wound Repair Regen* (total link strength = 22,430), and *J Invest Dermatol* (total link strength = 19,622) (Figure 5A).

For the co-citation analysis of cited authors, we screen the authors who are cited 15 or more and find 48 out of 17,979 authors, among which the five authors with the highest connection strength are cited “Wolcott, Rd (total link strength = 1,027)” “Dowd, Se (total link strength = 969)” “Gardner, Se (total link strength = 860)” “Grice, Ea (total link strength = 781)” “Lipsky, Ba (total link strength = 512)” (Figure 5B).

Co-citation analysis of cited references by analyzing the cited number reveals the relevance of different cited references. The minimum cited number of cited references is 15, and 25 cited articles are selected out of 23,807 cited articles, among which the five articles with the highest connection strength are “Dowd Se, 2008, Plos One, v3, doi 10.1371/journal.pone.0003326 (total link strength:165)” “Gardner se, 2013, Diabetes, v62, p923, doi 10.2337/db12-0771 (total link strength:145)” “Loesche M, 2017, J Invest Dermatol, v137, p237, doi 10.1016/j.jid.2016.08.009 (total link strength:139)” “Wolcott Rd, 2016, Wound Repair Regen, v24, p163, doi 10.1111/wrr.12370 (total link strength:138)” “James Ga, 2008, Wound Repair Regen, v16, p37, doi 10.1111/j.1524-475x.2007.00321.x (total link strength:133)” (Figure 5C).

### 3.6. Co-authorship analysis of author, institution, and country

Co-authoring analyses examine the interactions between scholars in a field of study. Because co-authorship is a formal form of intellectual collaboration between scholars. Therefore, it is important to understand how scholars interact with each other, including relevant authorial attributes such as affiliates and countries. With the complexity of research methods and theories, cooperation among scholars has become a common phenomenon. Indeed, collaboration between scholars can lead to improvements in research, and contributions from different scholars can contribute to clearer and richer insights.

**Figure 6:**
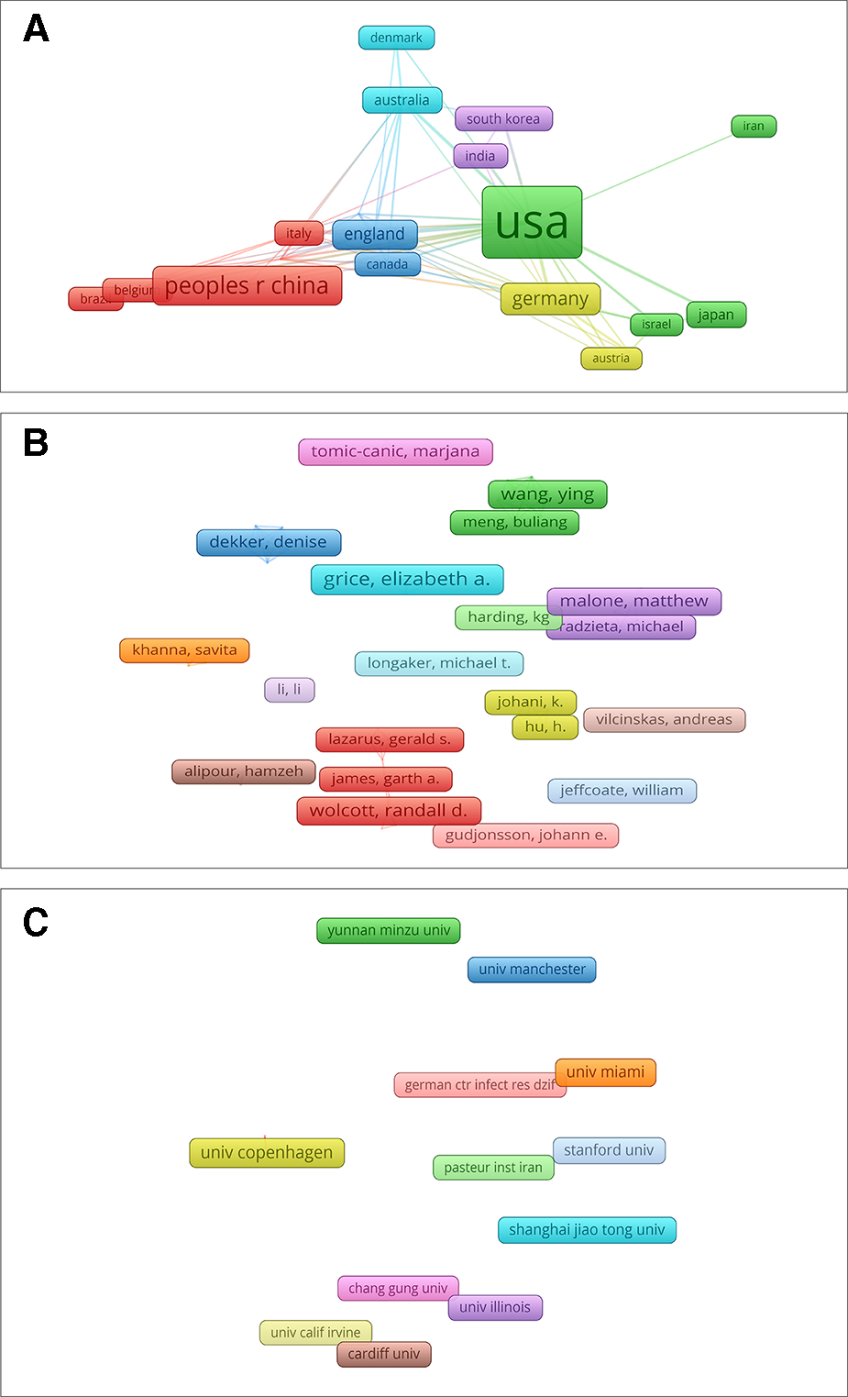
Mapping of co-authorship analysis about HTS technology associated with chronic wounds. (**A**) Mapping of the 20 identified countries. (**B**) Mapping of the 55 identified authors. (**C**) Mapping of the 30 identified institutions.

Regarding the co-authorship analysis of countries, we set the condition that there are more than 7 works in the country, and 20 of 61 countries meet the condition. The five countries with the strongest connections and influence are “United States (total link strength = 54)”, “England (total link strength = 32)”, “Germany (total link strength = 22)”, “Australia (total link strength = 18)”, and “Italy (total link strength = 15)”. It is worth mentioning that although China is second only to the United States in Np, it has weak cooperative relations with other countries, with a total link strength of only 4 (Figure 6A).

We used VOS viewer to conduct co-authorship analysis of the author and set the condition as the authors of at least 3 documents. A total of 55 authors were screened out. However, due to the small sample size, only 9 authors showed a strong co-authorship relationship. The first five authors are listed as follows: Yang Xinwang (total link strength = 23), Wang Ying (total link strength = 23), Li Xiaojie (total link strength = 20), Yang Meifeng (total link strength = 20), and Tang Jing (total link strength = 20) (Figure 6B).

When conducting co-authorship analysis of institutions, we set the condition that each institution has at least 5 works, and 30 out of 890 institutions meet the conditions after screening. Among them, the five most closely related institutions are “Western Sydney Univ (total link strength = 8)”, “Yunnan Minzu Univ (total link strength = 8)”, “Ingham Inst Appl Med Res (total link strength = 7)”, “Kunming Med Univ (total link strength = 7)”, and “Univ Penn (total link strength = 7)” (Figure 6C).

### 3.7. Co-occurrence analysis of keywords

The co-occurrence analysis of keywords can show the hot keywords in the research field and predict future research hotspots and trends (29). At the same time, the development trajectory and future trend of the field can be understood by analyzing the change in keywords over time (30). Keywords are words used more than 10 times in the title/abstract of all papers, selected and analyzed by VOS viewer.

**Figure 7:**
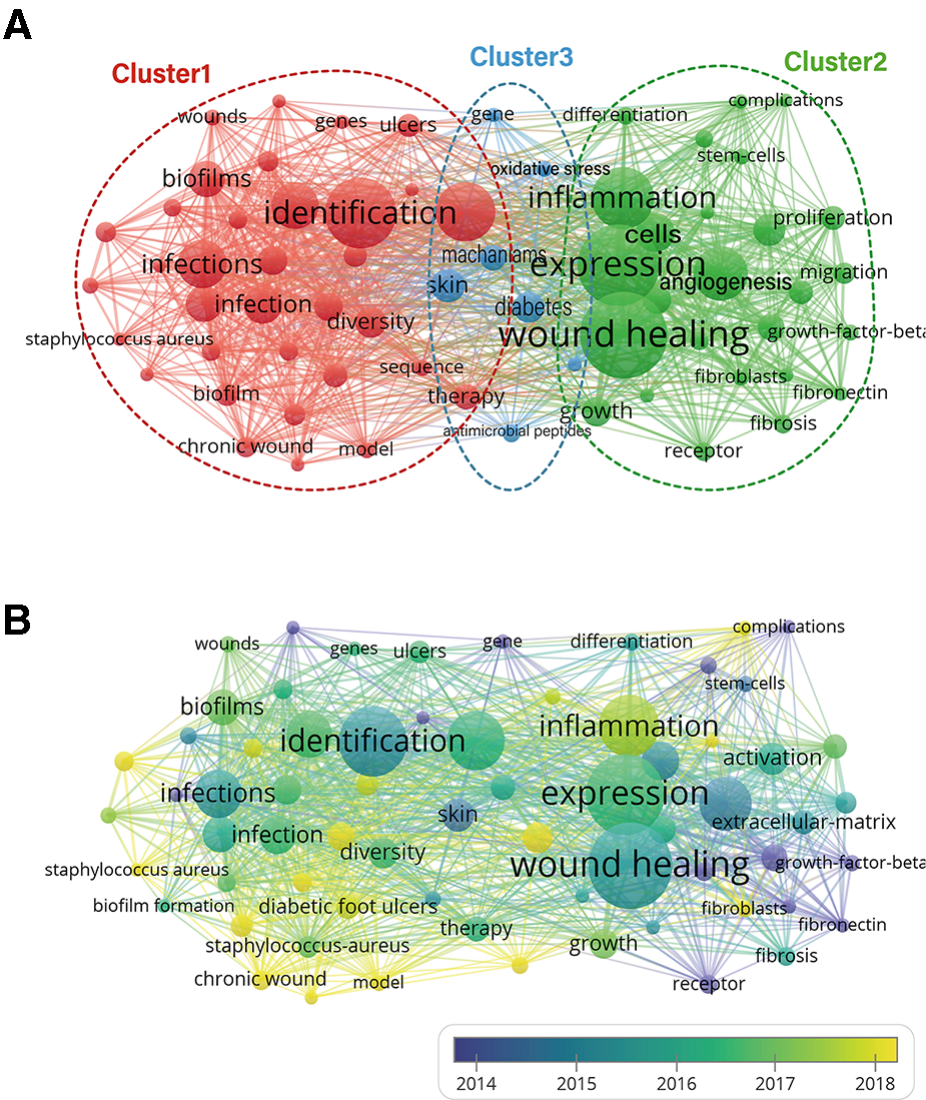
Mapping of co-occurrence analysis of HTS technology associated with chronic wounds. (**A**) Mapping of the 149 identified keywords. The frequency is represented by point size, and the keywords of research fields are divided into three clusters: microbial infection of chronic wounds (red), healing process of wounds and microscopic processes (green), skin repair mechanism stimulated by antimicrobial peptides and oxidative stress (blue). (**B**) Distribution of 149 identified keywords according to frequency and average time of occurrence; keywords in yellow appeared later than those in blue.

In addition to the searched words, a total of 67 keywords were frequently mentioned in the main text and abstract of 449 articles. These 67 keywords can be divided into 3 clusters. As seen from Figure 7A, Cluster 1 (red) is about microbial infection of chronic wounds such as diabetic foot. Cluster 2 (green) is about the healing process of wounds and microscopic processes such as cell expression and factor regulation in the inflammatory response, while Cluster 3 (blue) is about the skin repair mechanism stimulated by antimicrobial peptides and oxidative stress. The top 10 words with the highest frequency were wound healing (total link strength:256), expression (total link strength:241), inflammation (total link strength:190), identification (total link strength:179), chronic wounds (total link strength:177), bacteria (total link strength:162), infections (total link strength:150), and angiogenesis(total link strength:128), biofilms(total link strength:125) and diabetes(total link strength:119) indicate that the HTS of chronic wounds aims to study the identification of bacteria and microorganisms and the expression of inflammatory factors in the healing process of chronic wounds. The HTS of chronic wounds is a combination of basic and clinical research. In addition, the statistics of the average publication year (APY) of keywords are shown in Figure 7B. The color (from purple to yellow) represents the change in research hotspots of keywords. It can be seen that the latest five keywords are “chronic wound microbiota (APY = 2020.0)”, “staphylococcus aureus (APY = 2019.7)”, “fibroblasts (APY = 2019.0)”, “prevalence (APY = 2019.0)”, and “chronic wound (APY = 2018.6)”, which are recent research hotspots. The study of bacterial flora in chronic wounds is a new hotspot.

**Figure 8:**
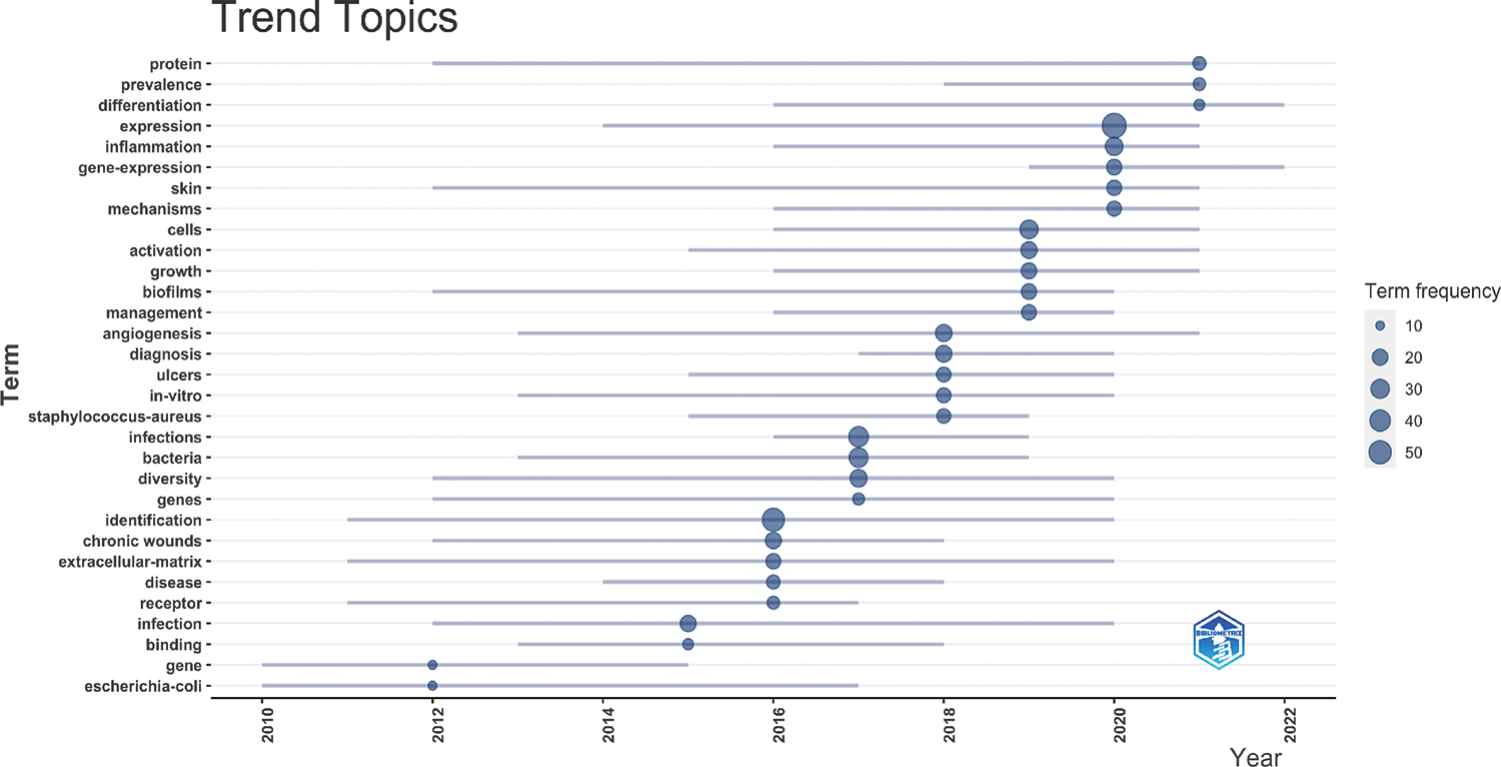
Global trend topic analysis of HTS technology associated with chronic wounds. Distribution of 31 identified topics according to average time of occurrence and frequency.

Then, we used bibliometriex to analyze the topic trend change from 2010 to 2022 and the duration of the popularity of related topics. As shown in Figure 8, topics with good performance in innovation and duration include “protein”, “expression”, “skin”, “biofilms” and “angiogenesis”. The basis of HTS research on chronic wounds is still related to several processes of wound healing. At the same time, the newly erupted topics are “prevalence”, “gene expression”, “inflammation” and “infection”. It can be seen that keywords related to HTS technology will be the popular trend of studying chronic wound healing in the future. The study of inflammation and bacteria in chronic wounds is a future trend (31).

### 3.8. Three-field analysis of keyword, country and journal

**Figure 9:**
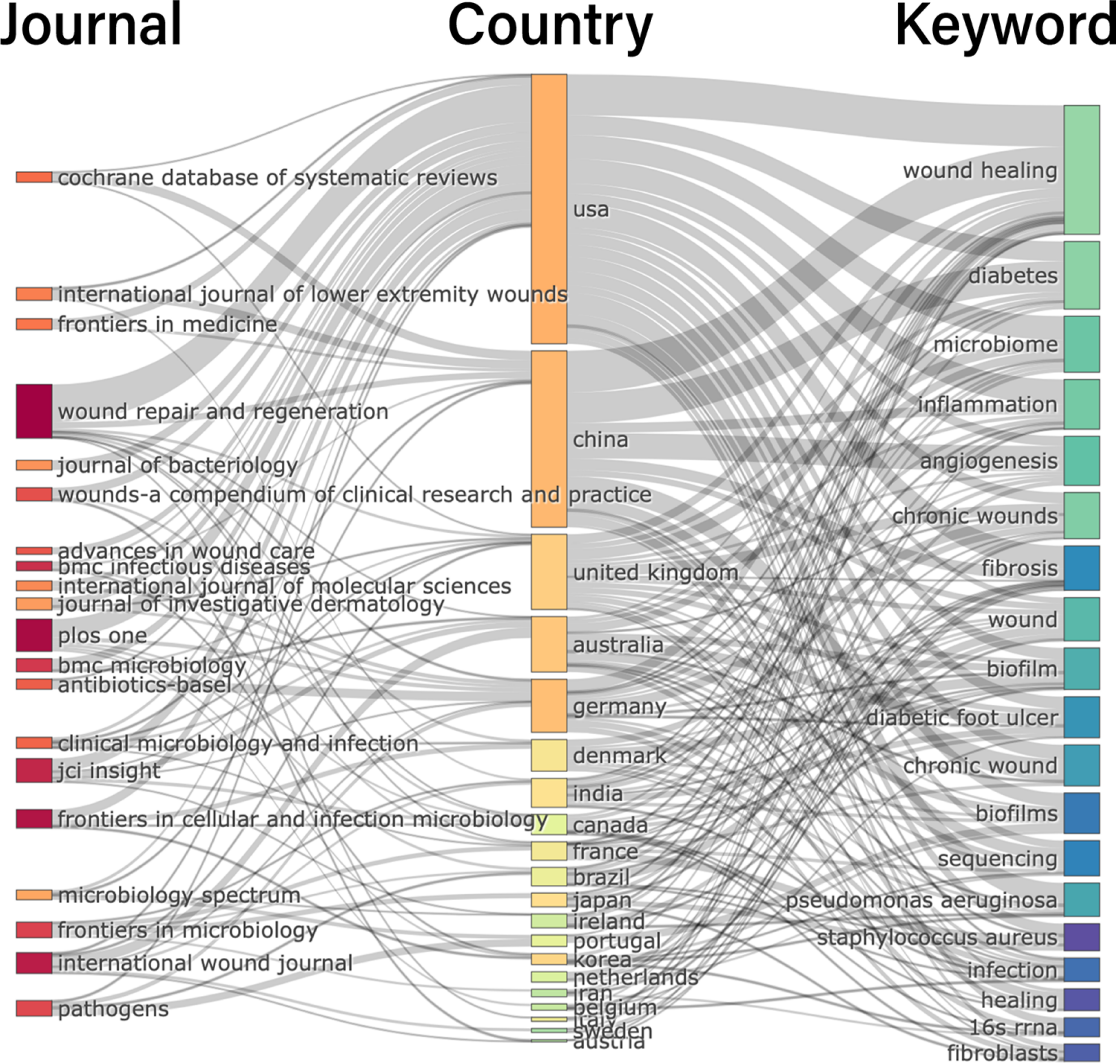
Three-field plot of analysis countries, keywords and journals about HTS technology associated with chronic wounds (middle field: countries, left field: journals, right field: keywords).

Meanwhile, by drawing the three-field plot of the top 20 keywords, country and journal, we also found different research priorities in different countries (Figure 9). The research trends in the area of HTS technology associated with chronic wounds were observed as follows: wound healing, diabetes, microbiome, inflammation, angiogenesis, chronic wounds, fibrosis, biofilm, sequencing, Pseudomonas aeruginosa, Staphylococcus aureus, infection, 16S rRNA and fibroblasts. In Western countries (mainly the United States, the UK and Australia), scholars focus more on the “microbiome”, “inflammation” and “biofilm” than in other countries (mainly referring to China). Chinese scholars show more interest in “diabetes”, “angiogenesis”, “chronic wounds”, and “sequencing”. We speculate that possible reasons include the large number of diabetic foot patients in China and the fact that scholars pay more attention to clinically relevant issues. However, Western scholars have paid more attention to the microscopic process and mechanism of inflammatory reactions and have proposed the concept of biofilms. Moreover, scholars in the United States prefer to publish their articles in the following journals: *Wound Repair and Regeneration*, *Plos One* and *Frontiers in Cellular and Infection Microbiology*. Scholars in China prefer to publish their articles in the following journals: Cochrane database of systematic reviews, international journal of low-extremity wounds and wound repair and regeneration.

## 4. Discussion

### 4.1. Trend of global publications

Delayed healing of chronic wounds (especially diabetic foot ulcers) has always been a hot topic in clinical research, among which the cellular ecology of chronic wounds and identification of bacterial pathogens have attracted increasing attention (32). Studies have shown that the microbiota of a wound can affect wound healing by either speeding up or slowing it down, depending on the type of microbiota on the wound (14, 15, 33, 35); therefore, it is necessary to decrease the cellular and molecular perturbations driving this abnormal wound healing state (35). HTS technology is a technology that emerged after 2000 and is increasingly applied in clinical and research (36). With the rapid development of this technology, it is possible to detect pathogens and monitor characteristic cell subsets (30, 37). HTS has several advantages: high sensitivity, the ability to detect more pathogens, the ability to identify rare bacteria, and the ability to identify dominant bacteria in mixed infections (38). In recent years, many researchers have published insightful literature in the field of HTS technology (39, 40). Furthermore, with the rapid development of second-generation sequencing technology, the scientific community has begun to increasingly use second-generation sequencing technology to solve biological problems (41–44). In terms of chronic wounds, the application of HTS technology to detect the distribution of wound microbiota and comprehensively understand the characteristics of wound microbiota to better guide clinical diagnosis and treatment has attracted the interest of an increasing number of scholars and has become a new research hotspot (14, 34). In this paper, we combined HTS technology with chronic wounds, especially diabetic foot wounds, to investigate the developmental trends and hotspots of research between the two fields from the Web of Science core collection database by using VOS viewer and Bibliometrix.

### 4.2. Quality and status of global publications

Among the top countries/regions, the United States ranked first and far ahead of second in both Np and Nc. This is closely related to America's global leading level of science and technology (45). The United States was the first country to master and try to use high-throughput technology sequencing. Most of the technology and related research was initially proposed by American scholars, and the United States went deeper in this field compared with the rest of the world (46, 47). In terms of Np, China was firmly in second place. It is worth mentioning that since 2010, China has only begun to publish articles in this field, and in the following years, the number of articles in this field has increased rapidly, indicating that an increasing number of Chinese scholars began to turn their attention to HTS technology to find solutions to the problem of chronic wound healing. However, the Nc of China only ranked fourth, far behind the United States, and the average citations per item ranked 14th in the world. This result may indicate that scholars and institutes in China should make more efforts on the quality of their papers in this field. In addition, we found that Switzerland and Romania did not have a high number of publications, but the average single citation volume of the articles was very high (ranked 1st and 3rd), indicating that the articles of these two countries were of high quality and were widely recognized in the medical field.

Among the top 10 authors with the most publications, 4 authors were affiliated with institutions in China, 3 authors were from Australia, 2 authors were from the United States and 1 was affiliated with institutions in Denmark.

Although the Np and the Nc of the United States ranked first, the author from the United States only ranked third in the top 10 published articles, while the author from China with the most published articles was LI X, whose H-index was also the highest (H-index = 9), indicating that Li X's output in this field was both quantitative and qualitative. The inclusion of Australian authors may be related to Australia's close external cooperation in this field (Figures 6, 7). Strengthening international cooperation is of great help to exploration.

### 4.3. Research focus on high-throughput sequencing technology associated with chronic wounds

The hotspots and research trend predictions of HTS technology associated with chronic wounds are mainly based on cooccurrence analysis (48, 49). The map of co-occurrence analysis of keywords showed three main directions of this field (Figure 7A). These three directions represent different focuses, from clinical to basic, from macro to micro, and from phenomenon to mechanism, comprehensively covering the key issues in this field. Three-field analysis of keywords, country and journal illustrates the differentiation of research focuses and journals among countries. Modern microbial ecology research of human skin is the basis of chronic wound microbial ecology research (50). Since HTS technology is relatively relaxed to culture conditions and is convenient to use, HTS technology primarily iterates microarray and fingerprinting techniques and has become the method of choice for understanding microbiome diversity, evolutionary patterns, and population structure (20). Recently, some studies using HTS technology to identify biofilms of chronic wounds have been published (51). Bacteria in chronic wounds have been observed to form biofilms, resulting in delayed healing. In mature biofilms, bacterial growth is slowed by a lack of nutrients, leading to bacterial resistance to antibiotics (11).

## 5. Limitations of this study

Although this paper has made a detailed bibliometric analysis of HTS technology associated with chronic wounds and put forward numerous innovative opinions for the future research trends of this field based on these data, there are still some limitations that should be mentioned. First, the selected database is only one Web of Science core collection, and the number of articles obtained is limited. However, this is a fresh subject, and the research in this paper can still be of great significance, which provides a direction for the following research direction and content. Second, all the articles included in this study are in English, and the book chapter, proceedings paper, correction, letter, meeting abstract, note, review and other types of articles are excluded. This may bias the results of our study, but we still have reason to believe that the impact of the excluded articles on the true results is relatively minor, and the articles we included are relatively sufficient to illustrate the problem. Third, for the traceability of the research, 2 coauthors (PY and MH) selected the same time point to retrieve the database articles, while the database articles were updated daily, which may leave out some valuable studies. Although coauthors selected the data separately, encountered problems were resolved by consulting with experts, ensuring the authenticity and reliability of the data.

## 6. Conclusion

In conclusion, this study is the first bibliometric analysis to scientifically and comprehensively analyze the global trend of HTS technology application associated with chronic wounds over the past 30 years. This paper compares the research hotspots and directions in this field globally from the perspectives of countries, institutions and authors, analyzes the trend of international cooperation, and reveals the future development direction of the field and research hotspots of great scientific research value. Future research efforts in this field should include the following aspects: HTS technology to assist in identifying the microbial ecology of chronic wounds, analysis of the microbiota of diabetic foot wounds, and search for targets to promote the healing of chronic wounds. Through this paper, we can further explore the value of HTS technology in chronic wounds to better solve the problem of chronic wounds.

## Data Availability

The original contributions presented in the study are included in the article/Supplementary Material, further inquiries can be directed to the corresponding author/s.
